# Strategic Cognitive Sequencing: A Computational Cognitive Neuroscience Approach

**DOI:** 10.1155/2013/149329

**Published:** 2013-07-08

**Authors:** Seth A. Herd, Kai A. Krueger, Trenton E. Kriete, Tsung-Ren Huang, Thomas E. Hazy, Randall C. O'Reilly

**Affiliations:** Department of Psychology, University of Colorado Boulder, Boulder, CO 80309, USA

## Abstract

We address strategic cognitive sequencing, the “outer loop” of human cognition: how the brain decides what cognitive process to apply at a given moment to solve complex, multistep cognitive tasks. We argue that this topic has been neglected relative to its importance for systematic reasons but that recent work on how individual brain systems accomplish their computations has set the stage for productively addressing how brain regions coordinate over time to accomplish our most impressive thinking. We present four preliminary neural network models. The first addresses how the prefrontal cortex (PFC) and basal ganglia (BG) cooperate to perform trial-and-error learning of short sequences; the next, how several areas of PFC learn to make predictions of likely reward, and how this contributes to the BG making decisions at the level of strategies. The third models address how PFC, BG, parietal cortex, and hippocampus can work together to memorize sequences of cognitive actions from instruction (or “self-instruction”). The last shows how a constraint satisfaction process can find useful plans. The PFC maintains current and goal states and associates from both of these to find a “bridging” state, an abstract plan. We discuss how these processes could work together to produce strategic cognitive sequencing and discuss future directions in this area.

## 1. Introduction

Weighing the merits of one scientific theory against another, deciding which plan of action to pursue, or considering whether a bill should become law all require many cognitive acts, in particular sequences [1, 2]. Humans use complex cognitive strategies to solve difficult problems, and understanding exactly how we do this is necessary to understand human intelligence. In these cases, different strategies composed of different sequences of cognitive acts are possible, and the choice of strategy is crucial in determining how we succeed and fail at particular cognitive challenges [3, 4]. Understanding strategic cognitive sequencing has important implications for reducing biases and thereby improving human decision making (e.g., [5, 6]). However, this aspect of cognition has been studied surprisingly little [7, 8] because it is complex. Tasks in which participants tend to use different strategies (and therefore sequences) necessarily produce data that is less clear and interpretable than that from a single process in a simple task [9]. Therefore, cognitive neuroscience tends to avoid such tasks, leaving the neural mechanisms of strategy selection and cognitive sequencing underexplored relative to the large potential practical impacts.

Here, we discuss our group's efforts to form integrative theories of the neural mechanisms involved in selecting and carrying out a series of cognitive operations that successfully solve a complex problem. We dub this process strategic cognitive sequencing (SCS). While every area of the brain is obviously involved in some of the individual steps in some particular cognitive sequences, there is ample evidence that the prefrontal cortex (PFC), basal ganglia (BG), and hippocampus and medial temporal lobe (HC and MTL) are particularly important for tasks involving SCS (e.g., [10–14]). However, exactly how these brain regions allow us to use multistep approaches to problem solving is unknown. The details of this process are clearly crucial to understanding that process well enough to help correct dysfunctions, to better train it, and perhaps to eventually reproduce it in artificial general intelligence (AGI).

We present four different neural network models, each of a computational function that we consider crucial for strategic cognitive sequencing. The first two models address how sequences are learned and selected: how the brain selects which of a small set of known strategic elements to use in a given situation. The first, “model-free learning,” is a model of how dopamine-driven reinforcement learning in the PFC and BG can learn short cognitive sequences entirely through trial and error, with reward available only at the end of a successful sequence. The second, “PFC/BG decision making” (PBDM), shows how cortical predictions of reward and effort can drive decision making in the basal ganglia for different task strategies, allowing a system to quickly generalize learning from selecting strategies on old tasks to new tasks with related but different strategies. The last two models apply to selecting *what* plans or actions (from the large set of possibilities in long-term semantic memory) will be considered by the two “which” systems. The third model, “instructed learning,” shows how episodic recall can work with the PFC and BG to memorize sequences from instructions, while the last “subgoal selection” model shows how semantic associative processes in posterior cortex can select representations of “bridging states” which also constitute broad plans connecting current and goal states, each of which can theoretically be further elaborated using the same process to produce elaborate plan sequences.

Because these models were developed somewhat separately, they and their descriptions address “actions,” “strategies,” “subgoals,” and “plans.” We see all of these as sharing the same types of representations and underlying brain mechanism, so each model actually addresses all of these levels. All of these theories can be applied either to individual actions or whole sequences of actions that have been previously learned as a “chunk” or plan. This hierarchical relationship between sequence is well understood at the lower levels of motor processing (roughly, supplementary motor areas tend to encode sequences of primary motor area representations, while presupplementary motor areas encode sequences of those sequences); we assume that this relationship holds to higher levels, so that sequences of cognitive actions can be triggered by a distributed representation that loosely encodes that whole sequence and those higher-level representations can then unfold as sequences themselves using identically structured brain machinery, possibly in slightly different, but parallel brain areas.

Before elaborating on each model, we clarify the theoretical framework and background that have shaped our thinking. After describing each model, we further tie each model to our overall theory of human strategic cognitive sequencing and describe our planned future directions for modeling work that will tie these individual cognitive functions into a full process that learns and selects sequences of cognitive actions constituting plans and strategies appropriate for novel, complex mental tasks, one of humans' most impressive cognitive abilities.

## 2. Theoretical Framework

These models synthesize available relevant data and constitute our attempt at curren best-guess theories. We take a computational cognitive neuroscience approach, in which artificial neural network models serve to concretize and specify our theories. The models serve as cognitive aids in a similar way to diagramming and writing about theories but also serve to focus our inquiries on the computational aspects of the problem. These theories are constrained not only by the data we specifically consider here but also by our use of the Leabra modeling framework [15, 16]. That framework serves as a cumulative modeling effort that has been applied to many topic areas and serves to summarize a great deal of data on neural function. This framework serves as a best-guess theory on cortical function, and individual models represent more specific, but still empirically well-supported and constrained theories of PFC, basal ganglia, reward system, and hippocampal function. Here, we extend these well-developed theories to begin to address SCS.

We also take our constraints from purely cognitive theories of cognitive sequencing. Work on production system architectures serves as elaborate theories of how human beings sequence cognitive steps to solve complex problems [17–19]. The numerous steps by which a production system model carries out a complex task such as air traffic control [20] are an excellent example of cognitive sequencing. Our goal here is to elaborate on the specific neural mechanisms involved, and in so doing, we alter those theories somewhat while still accounting for the behavioral data that has guided their creation.

Neural networks constitute the other class of highly specified and cumulative theories of cognition. However, these are rarely applied to the type of tasks we address here, in which information must be aggregated from step to step, but in arbitrary ways (e.g., first figure out center of a set of points, then calculate the distance from that center of points to an another point, and then based on that distance, estimate the likelihood that the point shares properties with the set). This is essentially because neural networks perform information processing in parallel and so offer better explanations of single-step problem solving. Indeed, we view humans' ability to use strategic cognitive sequences as an exaptation of our ancestral brain machinery, one that makes us much smarter by allowing us to access a range of strategies that lower animals largely cannot use [21, 22].

Because of the weaknesses in each approach and the paucity of other mechanistically detailed, cumulative models of cognition, we take inspiration from the well-developed theories from production systems about how cognitive steps are sequenced [17–19, 23] while focusing on artificial neural network-centered theories on the specifics of how individual cognitive actions are performed. This perspective is influenced by prior work on hybrid theories and cognitive architectures based on ACT-R and Leabra networks for a different purpose [24]. ACT-R [18] is the most extensively developed production system architecture and the one which most explicitly addresses physiology, while Leabra is arguably the most extensively developed and cumulative theory of neural function that spans from the neural to cognitive levels.

In ACT-R, the sequence of cognitive actions is determined by which production fires. This in turn is based upon the “fit” between the conditions of each production and the current state of the cognitive system (which also reflects the state of the environment through its sensory systems). This function has been proposed to happen in the basal ganglia (BG) [25, 26], and this has been borne out through matches with human neuroimaging data [25]. While it is possible that the BG is solely responsible for action selection in well-practiced cases [27], we focus on the learning process and so on less well-practiced cases. In our neural network framework, we divide this functionality between cortical and BG areas, with the cortex (usually PFC) generating a set of possible cognitive actions that might be performed next (through associative pattern matching or “constraint satisfaction”), while the basal ganglia decides whether to perform each candidate action, based on its prior relationship to reward signals in similar circumstances.

In modeling this process, we draw upon previous work from our group in modeling the mechanisms and computations by which the PFC and BG learn to maintain useful information in working memory [28–32]. The prefrontal cortex basal ganglia working memory (PBWM) models developed by O'Reilly and colleagues integrate a wealth of electrophysiological, anatomical, and behavioral data, largely from animal work. Working memory also appears to be a large component of executive function, because in many cases a specific task is performed by virtue of maintaining an appropriate task set [33], in effect remembering what to do. Those maintained representations bias other brain processing through constraint satisfaction. Because it explains the deep question of how we learn our executive function (EF), this theory makes progress in dispelling the “homunculus” [30], by explaining how complex cognitive acts are performed by a collection of systems, each of which supplies a small part of the overall intelligence, decision making, and learning.

In essence, the PBWM framework extends the wealth of knowledge on the role of the basal ganglia in motor control to address working memory and executive function. This is possible because there are striking regularities across areas of frontal cortex, so that the anatomy of cortex and basal ganglia that subserves motor function is highly similar to prefrontal and anterior BG areas known to subserve WM and EF [34]. This anatomy is thought to help select potential motor actions by “gating” that information through thalamus back to cortex, amplifying it and so cleanly selecting one of the several possible candidate actions represented in the cortex (e.g., [35]). The core hypothesis of PBWM is that these same circuits help select which representations will be actively maintained in PFC by fostering local reverberant loops in the cortex, and between cortex and thalamus, and by triggering intrinsic maintenance currents that enable self-sustained persistent firing in cortical pyramidal neurons. The reinforcement learning mechanisms by which BG learns which actions are rewarding also apply to learning what to remember and so what to do.

The primary value and learned value (PVLV) model of dopamine release as change in reward prediction [36, 37] is also a key component of PBWM and is in turn based on electrophysiological and behavioral data from a collection of subcortical areas known to be involved (e.g., [38–41]). The known properties of dopamine release indicate that it serves as a reward prediction error signal [42] which has informational properties that make it useful for driving learning [43, 44]. This system learns to signal when a new set of representations will likely lead to reward in a biologically realistic variant of the function of the better-known temporal difference (TD) algorithm when it is supplemented with “eligibility trace” information (e.g., [45]). This reward prediction function is crucial, because the difficulty in assessing the benefit of an action (whether it be cognitive or behavioral) is that the actual reward achieved by that action very often occurs later in time and so cannot be used directly as a learning signal [46, 47]. Instead, the system learns to perform actions that are predicted to gain reward. This reinforcement learning trains the striatum and works alongside the more powerful associative and error-driven learning within the PFC portion of PBWM that learns the representations (and therefore the associative semantics) of candidate actions to take.

In the remainder of the paper, we present an overview of four models that elaborate on this process in several ways. The first addresses how the learning mechanisms described previously and elaborated upon in works by various workers in our group [36, 37, 48, 49] can learn short sequences of cognitive actions, when they are sequentially dependent and so must be performed in the right order to achieve reward. The second describes how the hippocampus can achieve instructed learning, participating in the constraint satisfaction process of deciding which action to consider performing, as when we perform a novel task based on memorized instructions. The third model considers how slow, cortical associative learning can contribute to that same “which” process by using constraints of the current state and the goal to arrive at a subgoal that can serve as a viable next step in the sequence. Finally, we close with some discussion of the state of this research and the many remaining questions.

## 3. Model-Free Reinforcement Learning

Model-free reinforcement learning (RL) can be defined at a high level as learning which actions (which we take to include cognitive “actions”) produce reward, without any other knowledge about the world [43]. While the learning mechanisms we describe here are purely trial and error, the same learning mechanisms apply to model-driven or “hypothesis-driven” learning as well. For instance, the same learning principles apply when using actions, explicit plans from memorized instructions, or semantic associations as outlined in the final two models we describe later.

In hopes of better understanding how this process could occur in neural tissue, we have leveraged the prefrontal cortex basal ganglia working memory framework, or PBWM [28–32]. Under this account, a basic actor-critic architecture [43, 50] naturally arises between the prefrontal cortex (PFC), the basal ganglia (BG), and the midbrain dopamine system as modeled by our PVLV system described previously. PVLV serves as the critic, evaluating the state of the network and providing dopamine bursts or dips for better than and worse than expected outcomes, respectively. The BG system is naturally situated to perform the functions of the actor based on its known role in selecting motor actions (and by hypothesis, selecting cognitive actions with analogous neural mechanisms in more anterior regions of PFC). Using the critic's input, the BG learns from experience a policy of updating segregated portions of the PFC as task contingencies change. The PFC is able to maintain past context and provides a temporally extended biasing influence on the other parts of the system. It is helpful to view this entire process as a “gating” procedure: the BG gating controls that are being actively maintained within the PFC, and therefore subsequently biasing (controlling processing in) other cortical areas. When the gate is closed, however, the contents of the PFC are robustly maintained and relatively protected from competing inputs. Importantly, as task contingencies change and the actor determines that a change is needed, the gate can be opened allowing new, potentially more task appropriate, content into the PFC.

**Figure 1:**
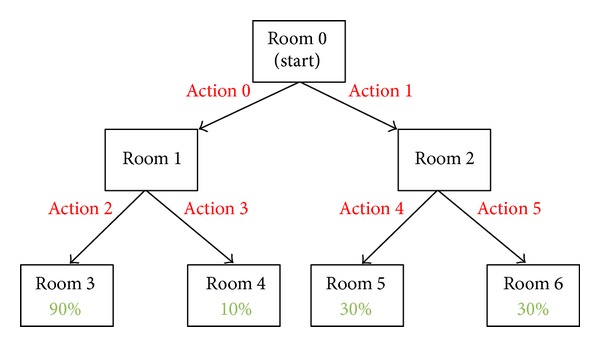
Simple state-based room navigation task. The percentages of the last level of rooms at the bottom of the figure represent the probability that the agent will get rewarded if it chooses the path that leads to the respective rooms.

The simple RL-based learning of the PBWM framework allows us to easily and naturally investigate one manner in which the brain may be capable of utilizing model-free RL in order to solve a simple task. In short, the network must learn to maintain the specific actions taken and evaluate this sequence based on either the success or failure of a simulated agent to attain reward. The simple example task we use is a basic state-based navigation task (abstracted at the level of “rooms” as states) in which a simulated agent must navigate a state space with probabilistic rewards as inspired by the work of Fu and Anderson [51] (see Figure 1). The goal of the task is simply to learn an action policy that leads to the highest amount of reward. To achieve this, the agent must make a choice in each room/state it visits to move either to the next room to the left or the next room to right but always moving forward. The only rooms that contain reward are at the final level (as in most tasks). The structure of the reward is probabilistic, so a single room is the most consistent provider of reward (Room 3 in Figure 1), but the others have a lower chance to be rewarding as well. In order for the PBWM framework to ultimately succeed, it must be able to maintain a short history of the actions it took and reward or punish these action choices in the final presence or absence of reward. This is a simple task, but a learning in this way is a valuable tool when the system must learn basic actions first in order to succeed at more extensive cognitive sequencing tasks.

### 3.1. Description of the Model

**Figure 2:**
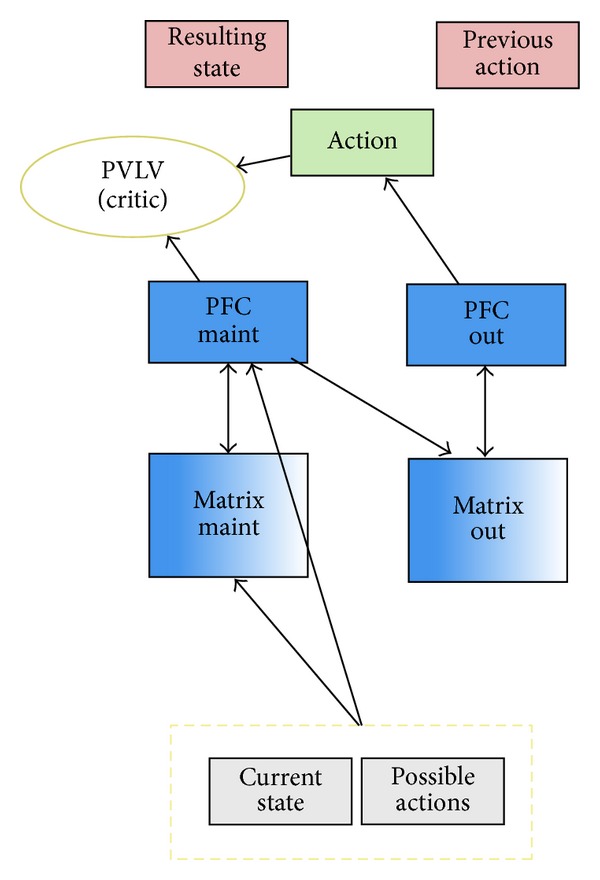
Model-free network architecture. Based on both current state and possible actions, the “matrix maint” determines what to maintain in PFC. Based on the stored information in PFC, “matrix out” determines the next chosen action via PFC out. PVLV (consisting of multiple biological systems) evaluates the actions (critic) and helps train the model. See text for in-depth description and functions of the various components of the network. Detailed network architecture is highly similar to the PBDM model discussed later.

The model-free RL network is depicted in Figure 2. The ultimate goal of the network is to receive reward by determining the best action to take given the reward structure of the simulated environment. There are many models of reinforcement learning in similar domains, and the PBWM and PBDM models have been applied to learning in superficially similar domains. However, some very important differences make the setup of this model unique. Most importantly, the final outcome (what Room the network ends up in based on the action chosen) of the network is not determined in the standard neural network manner of having activation propagate through units and having a competition that determines the winner. Instead, the network chooses an action via the *action* layer, which is the only traditional output layer in the network. The possible actions can be thought of as any atomic action that may result in a change of state, such as “go left” or “go right.” After the network chooses an action, a state transition table is used to determine the outcome of the action. More specifically, the network makes a decision about what action to take, and program code determines what the effect is on the environment of the simulated agent. The outcome is reported back to the network via the *resulting state* layer, but for display purposes only (not used in any computation). The example trial stepped through later in this section will help to clarify this process.

#### 3.1.1. Network Layer Descriptions

- Action layer: this is the output of the network. The chosen action is used via a state transition table to choose a new room. In the current simulation, the room choice is completely deterministic based on the action.
- CurrentState layer: this is a standard input layer. The CurrentState is the current state (room) that the model is occupying.
- PossibleActions layer: this is the second input layer. The layer is used to specify what “legal” actions are based on the current state that the network is occupying. Importantly, PossibleActions provides the main signal to the simulated basal ganglia to determine the gating policy, as well as the main input to the PFC. This ensures that only legal actions should be chosen (gated) at any given time.
- PreviousAction layer (display only): this is a display only layer. It maintains the last action choice that the network made. This can be useful to understand how the network arrived to its current state.
- ResultingState layer (display only): this is a display only layer. The ResultingState is the “room” that the simulated agent will arrive in based on the action that the network produced. The final room is used to determine if the agent should receive reward.
- PVLV layers: the PVLV layer(s) represents various brain systems believed to be involved in the evaluative computations of the critic [36].
- PFC maint and PFC out: simulated prefrontal cortex layers, the maint PFC is used to actively maintain information overextended delay period. The PFC out layer models the process of releasing this information, allowing it to affect downstream cortical areas and drive actual responses.
- Matrix maint and matrix out: these layers are used to model the basal ganglia system and represent the actor portion of the network. They learn to gate portions of the PFC, through experience, using the information provided from the PVLV system.

#### 3.1.2. Task Example

1. The current state (room) is presented to the network via the CurrentState layer. The inputs correspond to different rooms as shown in Figure 1, where Room 0 corresponds to the first unit in CurrentState layer, Room 1 to the second, Room 2 to the third, and so forth.
2. Using the CurrentState and the actions maintained within the PFC, the network must decide to go to the room to the left or the room to the right. This decision is reflected by activation in the action layer.
3. The action that is chosen by the network is used to determine where the simulated agent is in the current state space, and this is accomplished using a standard transition table to look up the next room. The actions are deterministic and move the agent directly to the room based only on the action.
4. The resulting state of the agent is returned to the network via activation in the CurrentState layer indicating the result of the action. Return to Step 2 unless the agent reaches a terminal room.
5. If the room reached by the agent is a final room, the reward probabilities for that room are used to determine the likelihood of reward to the agent.
6. Repeat from Step 1 until task is reliably learned.

### 3.2. Results

The network is capable of quickly learning the optimal policy of action sequences that optimize its reward on this task. To assess the ability of the network to solve this task, we set up a testing structure which allowed the network 75 “chances” to solve the task per epoch (block). At the end of the epoch, the average rate of reward was recorded for the simulated agent. This was repeated until either the agent received an average reward greater than 85% of the time or for 25 epochs (blocks), whichever came first. Ten simulated agents were ran, and 8 out of the 10 reached criteria of 85% average reward within 25 epochs. On average, it took 4 epochs to achieve this feat. While this may not appear to be a surprising result, the complex nature of the biologically realistic network made this far from a forgone conclusion. Indeed, many insights were gained about the nature of how the actor must balance its exploring of the state space with gaining reward. If the network randomly gets reward in one of the low-reward states, it must still be willing to explore its environment in order to confirm this finding. Conversely, if the network is in the high-reward state and does not receive reward, the (relative) punishment for this nonreward needs to allow a possible return to this same state at some point in the future in order to discover the optimal action policy. The limits of the framework are apparent in the 2 networks that did not reach criteria. In both of these cases, the agent randomly reached the low probability area of state space. In most cases, the agent is able to successfully explore other options and thus find the more rewarding rooms. However, the current PBWM framework will occasionally fail if reward is not present early enough in exploration process. We are investigating biologically inspired mechanisms to bootstrap the learning in more efficient ways. Encouraged by our initial framework, we are actively investigating how a simple model-free approach to learning basic sequences could be utilized by the human brain in order to scaffold up to more complex and interesting sequences. We are hopeful that concentrating on the relevant biological data and learning will provide us with useful insights to help us better understand how people are capable of such effortless sequencing of extended, diverse, and complex action plans.

We hypothesize that this type of learning aids in cognitive sequencing by allowing humans to discover useful simple sequences of cognitive actions purely by trial and error. While this learning does not likely account for the more impressive feats of human cognition, since these seem to require substantial semantic models of the relevant domain and/or explicit instruction in useful sequences, we feel that understanding what the brain can accomplish without these aids is necessary to understanding how the many relevant mechanisms work together to accomplish useful strategic cognitive sequencing.

## 4. Prefrontal Cortex Basal Ganglia Decision-Making (PBDM) Model

In the PBDM model, we primarily address decision making at the level of task strategies (task set representations in PFC, primarily dorsolateral PFC (DLPFC)). Decision making is important in many areas, but the selection of strategies for complex tasks is our focus. We believe that the same mechanisms apply to making decisions in many different domains.

The main idea behind PBDM is to computationally model the interactions between basal ganglia and medial prefrontal areas that represent particularly relevant information for making action plan or strategy decisions. Anterior cingulate cortex (ACC) and orbitofrontal cortex (OFC) serve as activation-based monitors of task affective value parameters [52, 53], including action effort in the ACC [54], and probability of reward in the OFC. These then project to the basal ganglia that controls updating in the DLPFC, giving it the necessary information to select choices in favor of lower effort and higher reward strategies. Because the ACC and OFC are themselves PFC areas with inputs from the same type of basal ganglia/thalamic circuits as motor and working memory areas, they are hypothesized to be able to rapidly update and maintain their value representations and, with a single gating action, change the evaluation to reflect new important information. This confers great flexibility and rapid adaptability to rapidly changing circumstances. Within this framework, several questions remain: what, more precisely, do the ACC and OFC represent? How can these representations drive appropriate gating behavior in the DLPFC BG? How are appropriate representations engaged in novel task contexts?

**Figure 3:**
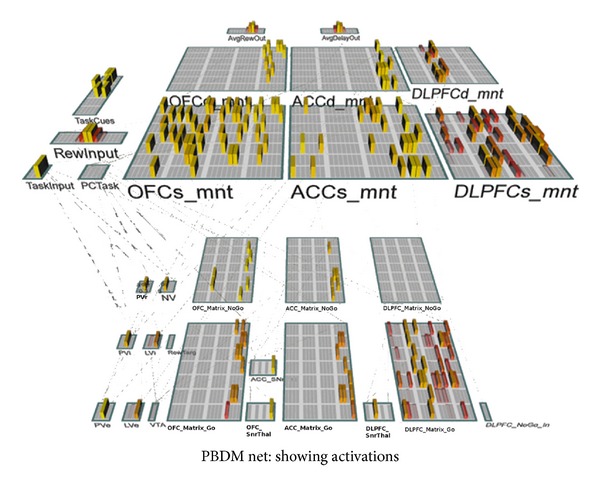
PBDM decision-making model. This figure shows the PBDM network and the components it models. The bars show the activation strengths of each of the units in the model for a particular point in time.

In the initial version of the PBDM model, described in more detail later and shown in Figure 3, we adopt simple provisional answers to these questions while recognizing that these likely underestimate the complexity of what happens in the real system. In particular, while ACC is often (and in our model) assumed to represent effort, its true role is more complex. The current state of knowledge on these issues is reviewed thoroughly by Kennerley and Walton [55]. The ACC and OFC in our model compute running time-averaged estimates of effort and reward probability, respectively, based on phasic inputs on each trial. If a task is ongoing, the ACC just increases its running average of time-effort by one. When a reward value is received or not (when otherwise expected), the OFC increments its running average estimate of reward probability. We have four different stripes within the ACC and OFC, each of which receives input from and so has a representation determined by one of the task strategies represented in the parietal cortex. These are thought of as very general strategies for dealing with spatial information, and over a lifetime of experience, we build up reasonable estimates of how effortful and rewarding they are on average in similar tasks. In order to in part capture the importance of context, there is also a randomly updated set of task features, which represent specific details about each different task that the model learns to perform. Over time, the model learns to pay attention to the ACC/OFC value representations in selecting a task strategy and pay less attention to these idiosyncratic task cues. Having done so, the model can then generalize to novel task contexts, by paying attention to the underlying spatial task values and ignoring the novel task features. Then, as the novel task progresses, actual experienced reward and effort drive the ACC and OFC representations, providing a more accurate picture for decision making going forward. This is the overall model we think applies to subjects as they engage in novel tasks with multiple possible strategies.

We conceptualize this PBDM process as engaging when people are actively and explicitly considering a new strategy or similar decision. We model an abstract spatial task, in which the strategies consist of individual spatial properties of groups of similar items. Strategies consist of considering one or a combination of these properties. There are 4 different strategies considered (listed by increasing order of both effort and reward probability; the precise values vary by task): Distance Only, Distance + BaseRate, Distance + Radius, and Distance + BaseRate + Radius. These are merely example strategies associated with a hypothetical spatial estimation task and are therefore sometimes also simply referred to as strategies 0 to 3, respectively; the task is not implemented for this model outside of entirely hypothetical probabilities of success (reward) and level of effort (time to implement). The weights for the PBDM component are trained to model a long history of experience with these hypothetical reward and effort values. After this learning (and purely through it), the OFC reward representations primarily bias the Go pathway, while the ACC effort representations bias the NoGo pathway. It is this balance between Go and NoGo that then ultimately determines the strategy selected. In our models, we observe that different random initial weights produce different individual preferences along this tradeoff.

The network performs various tasks (which switch every 10 trials during pretraining, simulating the intermixed variety of spatial tasks a person encounters during their daily life). The probability of reward and the number of trials required are determined by the selected strategy, the task representation that the DLPFC maintains. In reality, the possible strategies and therefore the representational space would be much larger, but we have narrowed it down to just 4 different states in a localist representation, (called Distance, Dist + Base Rate, Dist + Radius, and Dist + BaseRate + Radius; the original relation of these strategies to a particular task is irrelevant since the base task was abstracted to only the strategy component for this model). The inner loop per trial consists of “performing” the task in question, which happens through task-specific areas responding to the DLPFC task representation. We model that process here only at the most abstract level: each strategy takes an amount of time and has a probability of success that varies for each possible task. Thus, the PBDM network only experiences the overall feedback parameters: number of trials and probability of reward at the end of those trials. We do not model the process of carrying out these strategies; each of the models here could also be applied to understanding how a particular strategy unfolds into an appropriate sequence of cognitive actions.

The overall behavior is thus as follows: select a DLPFC task representation, run a number of blank trials (blank since we assume that the lower-level processes that carry out the strategy have little influence on this level of cortical machinery) according to the “effort” parameter (representing task performance), then receive reward with given probability determined by the PCTask representation that the DLPFC task representation drives, and then repeat. Over time, the BG gating units for the DLPFC are shaped by the effort/delay and reward parameters, to select DLPFC stripes, and associated reps that are associated with greater success and shorter delays.

The BG “Matrix” layer units control gating in DLPFC and so, ultimately, make final decisions on strategy choice. They receive inputs from the ACC and OFC, which learn over time to encode, using dynamic activation-based updating, running time averages of reward and effort, associated with the different strategies on the different tasks. Because we assume that mental effort is equal per unit time across strategies, the effort integration is identical to time integration in this case. Critically, because this is done in activation space, these can update immediately to reflect the current PCTask context. Over time, the BG learns weights that associate each OFC and ACC unit with its corresponding probability of success or effort. Thus, an immediate activation-based update of the ACC and OFC layers will immediately control gating selection of the DLPFC layers, so that the system can quickly change its decision making in response to changing task contexts [52, 56, 57].

Thus, the early part of the network training represents a developmental time period when the ACC and OFC are learning to perform their time-averaging functions, and the DLPFC BG is learning what their units/representations correspond to in terms of actual probability of reward and effort experienced. Then, in the later part, as the DLPFC task representations continue to be challenged with new task cue inputs (different specific versions of this task space), the learned ACC/OFC projections into DLPFC BG enable it to select a good task strategy representation on the first try.

### 4.1. Details of Network Layer Functions

- TaskInput: generalized task control information about the inner loop task being performed projects to DLPFC. We assume that this information comes from abstract semantic representations of the task at hand; this is likely represented in a variety of posterior and prefrontal regions, depending on the type of task. Use the following units/localist representations:
  - PERF—performing current task-signals that DLPFC should not update the task representation (see DLPFC NoGo In later); this repeats for the number of trials a given PCTask strategy requires and metes out the delay/effort associated with a given strategy.
  - DONE—done performing current task-reward feedback will be received in RewInput to OFC and PVe (PVLV); note that there is a “cortical” distributed scalar value representation of reward (RewInput), in addition to the subcortical one that goes directly into the reward learning system (PVe); conceptually these are the same representation, but their implementation differs.
  - CHOICE—DLPFC should choose a new task representation, based on influences from TaskCues, ACC, and OFC states; the newly gated DLPFC representation will then drive a new PCTask representation, which will then determine how many PERF trials are required and the probability of reward for the next DONE state.
- TaskCues: these are random bit patterns determined by the cur_task_no state, which drives DLPFC (both cortex and BG); they represent all the sensory, contextual, and instructional cues associated with a given specific task.
- PCTask reflects the actual task parameters. In this example, these are Distance, Dist + BaseRate, Dist + Radius, and Dist + BaseRate + Radius, but more generally this would represent a much larger space of task representations that have associated reward and effort parameters for different tasks. This may also reflect a combination of posterior cortical and also more posterior DLPFC representations that provide topdown biasing to these PC task representations and maintain them over shorter durations. The DLPFC in the model is the more anterior “outer loop” DLPFC that maintains higher-level, longer-duration task representations that are “unfolded” into useful sequences by other processes, including but likely not limited to those we address in the models here.
- RewInput: scalar val of reward input level activated during the DONE trial; this also has a −1 state that is activated whenever the network is in PERF task mode, and this is what triggers the incrementing of delay/effort in the ACC layer (i.e., both OFC and ACC feed off of this same basic RewInput layer, pulling out different information). This is overall redundant with signals in PVLV but packages them in a simple way for OFC/ACC to access and for us to manipulate for various experiments.
- OFC computes running time average of reward probability/magnitude; only updated when reward occurs (DONE trials), otherwise maintains the current estimate for PERF and CHOICE trials. The network learns coarse-coded distributed representation of this value, not in a scalar value format, through a “decoding” layer (AvgRewOut) that is in scalar value format. But it is the distributed representation that projects to DLPFC to bias its processing. It is not exactly clear what AvgRewOut corresponds to biologically, but the general idea is that there are autonomic level states in the brainstem, and so forth, that compute low-level time averages based on physiological variables (e.g., longer time average sucrose concentration in the blood), and that is what drives the OFC to learn to compute activation-based running time averages. See (vii) for the way this representation learns to affect DLPFC gating.
- ACC computes running time-average interval between reward trials which constitutes total effort on each task, since we assume roughly equal effort per time. It is updated on each performance trial and maintained during the DONE and CHOICE trials; each time step increases activation. As with OFC, this layer learns coarse-coded distributed representation of this value, not in a scalar value format, through a “decoding” layer (AvgDelayOut), which again reflects longer time-average metabolic cost variables.
- DLPFC encodes current task strategy and learns representations entirely through reinforcement learning stabilization. It receives information about each task from TaskCues; the Matrix layer also receives from ACC and OFC and learns over time to select task representations associated with good values of ACC and OFC (i.e., values of those that have been associated with rewards in the past). DLPFC also projects to PCTask, which in turn projects to ACC and OFC and “conditionalizes” (makes appropriate to the particular task) their representations.
- DLPFC_NoGo_In is our one “hack.” It turns on NoGo (strongly) whenever a task is being performed to ensure that the matrix does not update DLPFC midtask. This hard-coded behavior is simply the assumption that the DLPFC task set representation remains active during task performance; that is, people maintain one task set without switching strategies midway through more general learning: when you decide on a strategy, stick with it until you are done (or until it gets “frustrating” by consuming too much time).

### 4.2. Results

#### 4.2.1. Reward-Only Optimization: OFC Proof of Concept Test

**Figure 4:**
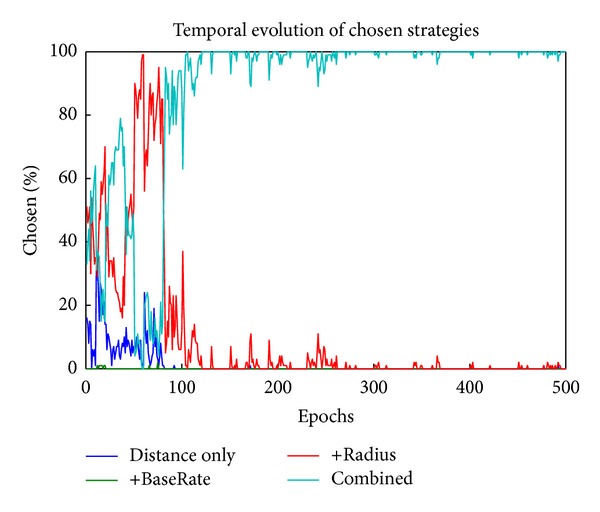
Developmental learning trajectory of PCTask selection. Early in learning it explores the different strategies, and later it learns to predominantly select the one (green line, strategy 3 (“Combined”)) that produces the best results.

**Figure 5:**
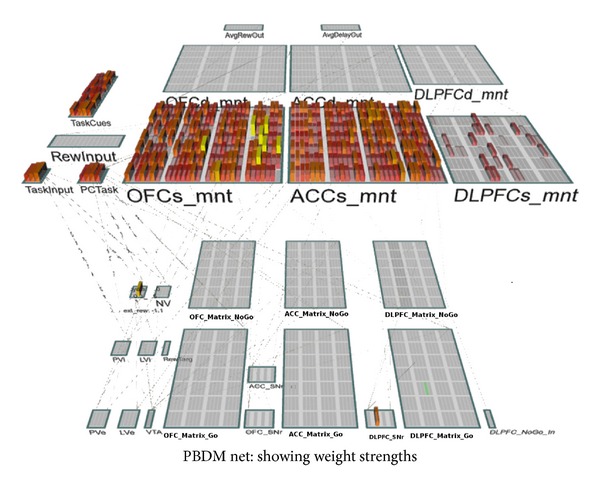
PBDM decision-making model. This figure shows the weights from the respective units to a unit in the DLPFC_Matrix_Go layer (green, lower right). It depicts the strength of weights towards the end of learning, at which point there are particularly strong connections from the core OFC distributed representations, which represent strategy's predicted reward value, established through learning.

The first proof of concept test sets the probability of reward to .2, .4, .6, and .8 for PCTask units 0–3, respectively (labeled “Distance only,” “+BaseRate,” “+radius,” and “Combined,” resp.), with delay set to a constant 1 trial (.2 parameter × 5 trials max delay) for all options. Thus, the best strategy is to select strategy 3, based on OFC inputs. As shown in Figure 4, the network does this through a period of exploration followed by “exploitation” of strategy 3, which is selected automatically and optimally immediately, despite changing TaskCues inputs. All of the batches (10/10) exhibited this same qualitative behavior, with a few stabilizing on strategy 2 instead of 3. This was the second-best strategy, and the fact that the model stabilized on this in some cases shows the stochastic process of sampling success that likely contributes to the selection of nonoptimal strategies in some real-life cases (since after the model stabilizes, it will not learn about potentially better strategies without some sort of external perturbation to force resampling). None stabilized on 0 or 1, since they have substantially lower reward probabilities. As shown in Figure 5, the weights into the Matrix Go stripe that gates DLPFC learned to encode the high-value OFC representations associated with the strategy 3 OFC representation.

#### 4.2.2. Delay-Only Optimization: ACC Proof of Concept Test

Next, we set probability to .6 for all strategies and set the delay factors to 1, 2, 3, and 4 trials of delay, respectively, for strategies 0–3. Without any PVLV feedback at all during the PERF trials, the network does appear to be sensitive to this delay factor, with strategy 0 (1 trial delay) being chosen preferentially. However, this preference is somewhat weak, and to produce stronger, more reliable preferences, we added a direct dopaminergic cost signal associated with delay, as has been shown empirically [58]. This modulation decreased the size of a DA reward burst in proportion to effort/delay (with a small weighting term). In our proof of concept test, this small modulation produced 50% of networks preferring the first (least delay) strategy.

#### 4.2.3. Balanced Reward and Delay (Actual Use Case)

**Figure 6:**
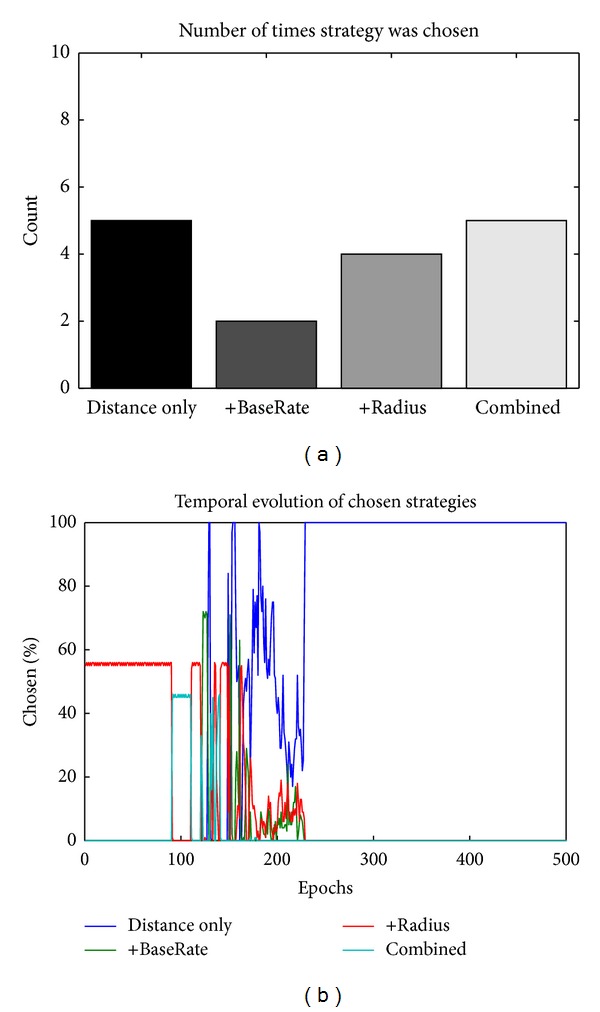
Balanced reward and delay. The left graph shows the number of times a strategy was chosen over 16 repeats with random initial weights, while the graph on the right shows the temporal evolution of selection for one randomly chosen network. The variability in the equilibrium strategy choice stems from the balance between reward and delay (the higher the reward, the higher the delay) making each strategy approximately equally rational to choose. As discussed in the reward-only case previously, the particular, random history of reward plays a large role in determining the ultimate strategy choice.

To simulate a plausible situation where there is a tradeoff between effort and reward, we set the reward factors to .4, .6, .6, and .8 and the delay factors to .2, .4, .6, and .8. This resulted in a mix of different strategies emerging over training across different random initial weights (“batches”) (proportions shown in Figure 6), with some preferring the low-effort, low-reward distance only option, while others going for the full Distance + BaseRate + Radius high-effort, high-reward case, and others falling in between. The particular results are highly stochastic and a product of our particular choices of reward and effort values; it is easy to push these preferences around by using different weightings of effort versus time. 

### 4.3. Discussion

The PBDM model shows how rapid updating in prefrontal cortex (as captured in the PBWM models and related work on persistent firing in PFC) can aid in decision making by allowing the system to use contextually appropriate representations of predicted reward and effort to drive decisions on task strategy. If the context (e.g., physical environment and task instructions) remains the same, then new learning in the ACC and OFC slowly updates the values of predicted reward and effort through weight-based learning. If, however, the context changes, representations in ACC and OFC will be “gated out,” so that a new set of neurons learns about the new context. Detailed predictions about the old context are thus preserved in the synaptic weights to that now silent units (because the learning rule we use, and most others, does not adjust weights to inactive neurons/units).

One way in which this preservation of contextually dependent ACC and OFC representations could be extremely useful is in interaction with episodic memory in the HC. We believe that predictive representations could also be retrieved to ACC and OFC from episodic memory in the hippocampus, a form of PFC-HC interaction similar to but importantly different from that we model in the “instructed learning” model.

This model primarily addresses the “strategic” component of strategic cognitive sequencing, but this type of effortful decision making, bringing the whole predictive power of cortex online to estimate payoff and cost of one possible sequence component, could help bootstrap learning through the mechanisms in either or both of the instructed learning and “model-free” models.

## 5. Instructed Learning

One source of complex, strategic cognitive sequences is learning them directly from instruction [59–61]. Humans have the remarkable ability to learn from the wisdom of others. We can take advice or follow instruction to perform a particular cognitive sequence. One such example may be observed daily by cognitive scientists who conduct human laboratory experiments. Most normal participants can well implement instructions of an arbitrary novel task with little or no practice. However, in the cognitive neuroscience of learning, reinforcement learning has been the central research topic and instructed learning appears to have been relatively understudied to date. In this section, we contrast reinforcement and instructed learning and outline the dynamics of instruction following in a biologically realistic neural model.

Reinforcement learning adapts behavior based on the consequences of actions, whereas instructed learning adapts behavior in accordance with instructed action rules. As a result, unlike the slow, retrospective process of trial and error in reinforcement learning, instructed learning tends to be fast, proactive, and error-free. In the brain, the neurotransmitter dopamine signals reward prediction errors for the basal ganglia to carry out reinforcement learning of reward-linked actions (for a discussion, see [62]). As for instructed learning, the human posterior hippocampus underlies verbal encoding into episodic memory [63] and use of conceptual knowledge in a perceptually novel setting [64].

Compared to reinforcement learning, instructed learning appears effortless. Why is learning so arduous in one mode but effortless in another? How exactly do we perform complex novel tasks on the first attempt? We propose that instruction offers nothing but a new plan of recombining old tricks that have been acquired through other forms of learning. In other words, instructions quickly assemble rather than slowly modify preexisting elements of perceptual and motor knowledge. For example, we can immediately follow the instruction: “press the left button when seeing a triangle; press the right button when seeing a square,” in which the action of button press is a preexisting motor skill, and visual recognition and categorization of shapes are also an already learned perceptual ability. Note also that understanding the instruction requires a previously learned mapping from language (e.g., the verbal command of “press”) to actual behavior (e.g., the motor execution of “press”). 

**Figure 7:**
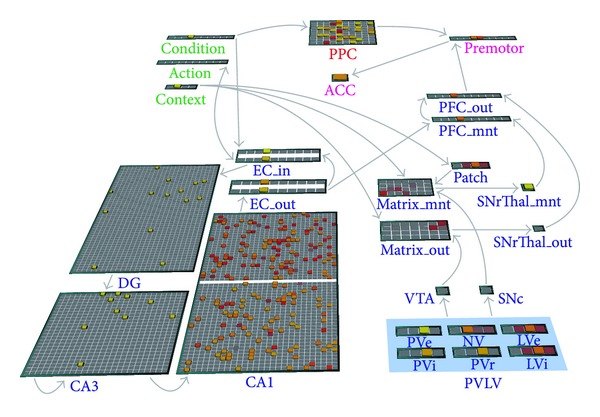
The instructed learning model. The model consists of two interactive learning pathways. The hippocampal-prefrontal pathway (i.e., lower part in the diagram) processes newly instructed conditional-action rules, whereas the parietal pathway (i.e., upper part in the diagram) processes habitual actions. The actions suggested by each of these pathways are then gated by the PFC portion.

To further study how instruction following is carried out from neural to behavioral levels, we constructed a model of instructed learning based upon known neuroanatomical and neurophysiological properties of the hippocampus and the prefrontal-basal ganglia circuits (Figure 7). Specifically, the model basal ganglia (BG) carries out reinforcement learning of motor execution (abstracted in the model to premotor); the model hippocampus rapidly encodes instructions as action episodes that can be contextually retrieved into the prefrontal cortex (PFC) as a goal for guiding subsequent behavior. Unlike a single-purpose neural network that slowly rewires the whole system to learn a new sensorimotor transformation, this general purpose instructable model separates motor from plan representations and restricts plan updating to lie within the fast-learning hippocampus, which is known to rapidly bind information into episodic memories.

**Figure 8:**
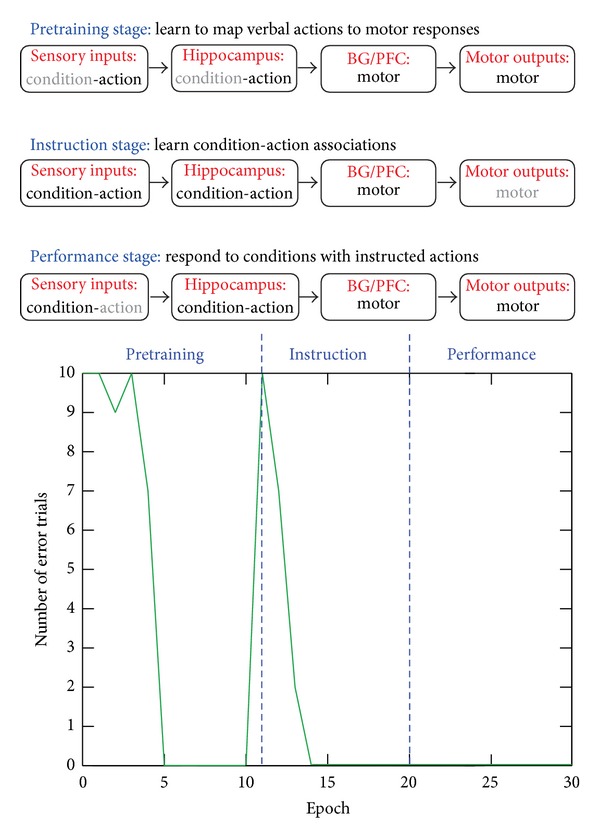
Instructed learning stages and simulation results. In the upper panel, black and grey texts denote present and absent representations, respectively. In the bottom panel, each epoch consists of 10 trials. Note that no error is produced during the Performance stage, since the prememorized mappings can be recalled perfectly after four repetitions during the Instruction period.

As a concrete example, the proposed model is instructed with 10 novel pairs of if-then rules (e.g., if you see A, then do B) and evaluated for its success in performing conditional actions (e.g., do B) when encountering a specific condition (e.g., seeing A). In the model, each of the “Condition,” “Action,” and “Premotor” layers consists of 10 localist representations of conditions, verbal actions, and (pre-)motor outputs, respectively. The model is pretrained with action-to-motor mappings (i.e., from verbal commands to premotor responses) during the Pretraining stage and then trained with condition-to-action mappings (i.e., if-then rules) during the Instruction stage. Finally, during the Performance stage, it is tested with Condition-to-Motor mappings without any inputs from the “Action” layer. The simulation results are shown in Figure 8. The model quickly learns an if-then rule in just few trials during the Instruction stage, and without further practice, it makes no error in carrying out these instructions for response during the Performance stage, just as human subjects often do after being presented with clear instructions and a short practice period.

 Inside the model, learning occurs in multiple parts of the architecture. During the Pretraining stage, the hippocampus learns to perform identity mapping for relaying information from the “Action” layer to the corresponding motor representations in the PFC layers. Meanwhile, BG learns to open the execution gate for PFC to output a motor decision to the “Premotor” layer. During the Instruction stage, the hippocampus associates inputs from the “Condition” and “Action” layers and learns each condition-action pair as a pattern. During the Performance stage, all the model components work together using mechanisms of pattern completion, and the hippocampus recalls instructions about what action to do based on retrieval cues from the “Condition” layer, and its downstream PFC either maintains a retrieved premotor command in working memory when BG closes the execution gate or further triggers a motor response in the “Premotor” layer when BG opens the execution gate.

Compared to earlier work on instructable networks [65], our model further explicates how different parts of the brain system coordinate to rapidly learn and implement instructions. Albeit simple, our instructed learning mechanisms can support strategic cognitive sequencing in that a cognitive sequence can be constructed from an ordered set of instructed or self-instructed operations. Beside sequential behavior, the model is being extended to also explain the interactions between instructions and experience (e.g., [66–69]) in the context of confirmation bias and hypothesis testing. The modeled ability of the hippocampus to memorize specific contingencies in one shot undoubtedly contributes an important piece of our ability to learn complex goal-oriented sequences of cognitive actions. Beyond simply memorizing instructions given by others, it can also aid in “self-instructed” learning by remembering successful steps learned by trial and error or other means for assembly into new sequences.

## 6. Planning through Associative Discovery of Bridging States

We explore the idea that the active maintenance of long-term goals in the PFC can work in conjunction with a network's semantic knowledge to identify relevant subgoals and then use those individual subgoals in a similar manner to bias action selection in the present. One fundamental question motivates this research. Given some ultimate goal, possibly associated with explicit reward, how does the system identify subgoals that lead to the final goal? Our hypothesis revolves around semantics, that is, knowledge about how the world works. Our model uses this knowledge to perform constraint satisfaction by using active representations of the current state (where I am) and the desired goal (where I want to be) to associatively arrive at a representation of a subgoal that “bridges” between the two states. This subgoal can serve as the focus for a strategy or plan to achieve the larger goal.

### 6.1. Description of the Model

There is a tension that exists between the temporal sequencing over one or more subgoals versus a multiple constraint-satisfaction approach that does things all in one step. It seems clear that both can be involved and can be important. So, when does the brain do one versus the other? We have adopted the following heuristic as a kind of corollary of Occam's razor. In general, the brain will by default try to do things in a single time step if it can; as an initial hypothesis, we suspect that bridging over a single subgoal is probably about as much as can be done in this way. When no such plan exists, a more complex process of navigating the modeled task-space through stepwise simulations of intervening states can be undertaken; because this process is among the most complex that humans undertake, a model that does this in a biologically realistic way is a goal for future research. Thus, our initial objective here is to try to demonstrate a one-step constraint satisfaction solution to a simple three-state problem: current state and end state to subgoal (“bridging”) state.

**Figure 9:**
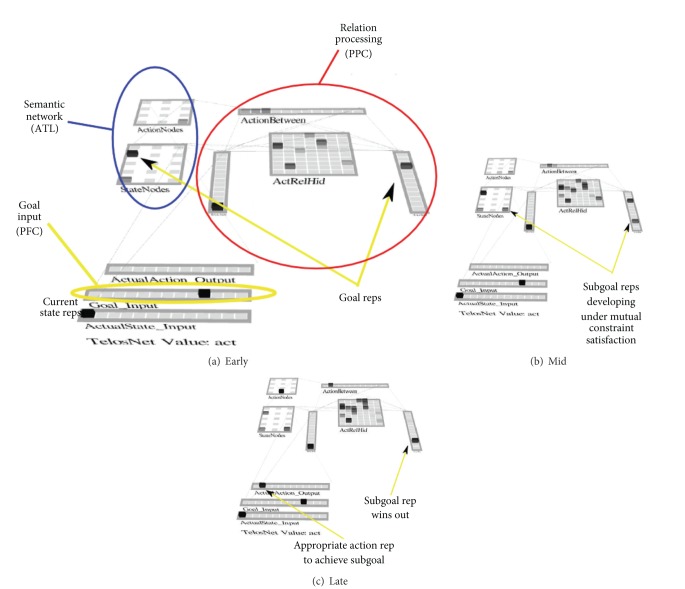
Subgoaling through constraint satisfaction. This figure shows settling of activations of the current state and goal state in both the *Semantic Network* (see text) and *Relation Area* (see text). (a) shows activations early in the settling process of a trial. (b) Activations midway into settling for a trial. The activation of two units in the rightmost Goal layer shows the constraint satisfaction process selecting two plausible subgoals. (c) Activations late in settling when they have converged. The network has settled onto the single most relevant subgoal through constraint satisfaction (simultaneous association from the current state and maintained goal state).

Another major issue is the tension that exists between state representations sometimes having to compete with one another (e.g., “What is the current state?,”) versus sometimes needing to coexist as in spreading activation so as to represent a full motor plan or model of state space (e.g., representing all three of the states in the previous three-state problem). The solution we have settled on is a division of labor between a relation processing area, possibly in the posterior parietal cortex (PPC, circled in red in Figure 9), and a semantic association area, possibly in the anterior temporal lobe (ATL, circled in blue). Because many brain areas are involved in semantics, the precise areas can be expected to vary with the semantic domain, but the mechanisms we describe are expected to be general across those variances. Figure 9 later illustrates these two distinct areas. The PFC (not explicitly modeled) is envisioned to represent the goal state and thus to bias processing in these two areas. The relation processing area is based on the ideas described in “Semantic Cognition” by Rogers and McClelland [70].

Training: the network is trained on the semantics of the State-Action-State triad relation (parietal/anterior temporal cortex) but includes connections to the semantic part of the network. The idea is that the relation area will learn the specific role relations between the states (before, after) and the actions (between states), while the semantic area will learn simple associations between the nodes. The former is dominated by a tight inhibitory competition, while the latter is more free to experience spreading activation. In this way, pre-training on all the individual S-A-S relations enables the bridging over an intermediate subgoal state and biases the correct action in the current state, under the biasing influence of the goal state.

As illustrated in Figure 9(a), which shows a network trained only on pairwise transitions between adjacent states, when a current state and a remote goal state are input, both are activated in both the semantic network and relation engine early in settling. At this very early stage of settling, there are three action units active in the ActionBetween layer (Relation Engine), which are all of the possible actions that can be taken in the current state (S0). Later in settling (Figure 9(b)), a third state unit comes on, which is the intermediate state between the current state and the goal. It becomes the only active unit due to a constraint satisfaction process that includes both bottom-up input from the current state and top-down input from the goal state. This in turn drives the intermediate state unit ON in AfterState layer in the RelationEngine module.

Finally, late in settling (Figure 9(c)), the intermediate state outcompetes the goal unit in the AfterState layer due to the attractor associated with the prior training of contiguous state transitions. This is associated with the third action unit in the ActionBetween and ActionNodes (Semantic Network) layers. This is the correct answer. This model illustrates how constraint satisfaction to find bridging states can work as one component of more complex planning.

### 6.2. Discussion

Subgoals in this context are conceived as a version of “cold” goals, defined as teleological representations of a desired state of the world that, in and of itself, does not include primary reward. Thus, in a sense, cold goals (here subgoals) are “just a means to an end.”

In thinking about the role of subgoals, a number of important issues can be identified. First, as already noted, a fundamental issue concerns how brain mechanisms create useful subgoals, if they are not provided externally. In addition, a second important issue is whether there are one or more biologically plausible mechanisms for rewarding the achievement of subgoals. This in turn has two subcomponents: (1) learning how to achieve subgoals in the first place (e.g., how to grind coffee in support of making coffee in the morning) and (2) learning how/when to exploit already familiar subgoal in the service of achieving a master goal (e.g., learning that having ground coffee is a precursor to enjoying a nice fresh cup of hot coffee for yourself and/or receiving kudos from your significant other). It is interesting to note that these two learning categories exhibit a mutual interdependence. Usually, learning how to achieve subgoals must precede learning to exploit them, although an interesting alternative can sometimes occur: if a learner is allowed to use its what-if imagination. For example, if a learner can do thought experiments like: “IF I had ground coffee, and cold water, and a working coffee maker, THEN I could have hot coffee.” Thinking about it over and over could transfer (imagined) value from the hot coffee to the ground coffee, and so forth, *which then* could be used as secondary reinforcement to motivate the learning of instrumental subgoals. This scenario-spinning behavior is not modeled in any realistic cognitive model of which we are aware; achieving this will be difficult but an important step toward understanding human intelligence.

A third critical issue is how subgoals are actually used by the system (in a mechanistic sense) in the service of pursuing the master goal. Here, the simple idea that serves as a kind of working hypothesis in our work is that the active maintenance of subgoals can serve to bias the behavior that produces their realization in a kind teleological “pull of the future” way. Finally, there then still needs to be some sort of cognitive sequencing control mechanism organizing the overall process, that is, the achievement of each subgoal in turn. Ultimately, in our way of thinking, this whole process can be biased by keeping the master goal in active maintenance throughout the process.

In sum, this model demonstrates a rough draft of one aspect of human high-level planning: abstract state representations allow constraint satisfaction processes based on associative learning to find a bridging state between current and goal states. We hypothesize that this process is iterated at different levels of abstraction to create more detailed plans as they are needed. However, we do not as yet have a model that includes the movement between different levels of plan abstraction. The other models presented here represent some of the mechanisms needed for this process but have yet to be integrated into a coherent, let alone complete, model of human planning.

Explaining how brains perform planning requires understanding the computational demands involved. The more abstract literature on the algorithmic and computational properties of planning in artificial intelligence research has thoroughly explored the problem space of many types of planning (e.g., [71–73]). Consistent with this proposed biological model, algorithmic constraint satisfaction solvers have been an important part of AI planning algorithms (e.g., [74, 75]). Other extensions and combinations of these models are also suggested by AI planning work; search-based algorithms (e.g., [76, 77]) show that sequencing, storing, and retrieval of state (as in the model-free and instructed sequencing model) are essential for flexible planning. We address some such possible combinations and extensions later.

## 7. General Discussion

The four models here represent an incomplete start at fully modeling human strategic cognitive sequencing. A full model would explain how basic mammalian brain mechanisms can account for the remarkable complexity and flexibility of human cognition. It would address the use of elaborate cognitive sequences which constitute learned “programs” for solving complex problems and how people generalize this ability to new problems by selecting parts of these sequences to construct appropriate strategies for novel tasks in related domains. A complete model is thus a long-term and ambitious project, but one with important implications for understanding human cognition.

The following primarily addresses the limitations in the work described and our plans to extend these models toward a more complete explanation of complex human sequential cognition. Although learning was integral to all presented models, demonstrating the feasibility of bootstrapping such flexible cognitive systems, the learning in these initial models was mostly still domain specific: models were trained within the class of tasks to be performed from a naive state. While the instructed model could generalise to a variety of unseen if-then rules and the constraint satisfaction model generalizes to unseen state-goal pairings, they were both only trained on their respective tasks.

In future work, we plan to extend this to a more sophisticated pre-training or scaffolding of networks that are more general and ecologically valid. Instead of beginning training of specific task structures from a naive network, the idea is to train the networks on a large variety of distinct tasks, progressing from simple to complex. The PBDM model, for instance, was trained in a relatively ecologically valid way but did not learn increasing complexity of tasks as it mastered simple ones as humans do. With increasing number of tasks trained, the network should learn to extract commonality between tasks, abstracting the essence of tasks into distinct representations. While it remains unclear what these task representations might look like on the finer biological scale, either from experimentation or computational modeling, it seems likely that representations for some basic computational building blocks of cognitive sequencing exist.

Such representations must, at an abstract level, include some of those found in any standard computer programming language, such as sequencing, loops, storing, and recalling of state. While the models presented here cannot accomplish any of these functions as they stand, we already have a rough basis for these basic task building blocks. All of the previous “program flow” functions can be seen as subsets of conditional branching (e.g., if you have not yet found the goal object, use a sequence that looks for it). The other models presented here (planning, model-free sequence learning, and decision making) address important aspects of how sequences are learned and used, but the instructed learning model alone is enough to understand one way in which the brain can exhibit such program flow control once a relevant sequence is learned. This behavior requires extending the model to store and use state information. This minor extension would include working memory updates in the potential actions and make action pairs conditional on those working memory representations as well as sensory inputs.

Dayan [78] has already explored this behavior in a more abstract version of PBWM. This model includes storage actions and dependency upon stored information consistent with the role for which PBWM was primarily developed, understanding how mechanisms evolved for gating motor actions control storage in working memory. Making memorized pairings dependent upon state information in working memory is also straightforward, and known basal ganglia connectivity suggests such a convergence of information between prefrontal working memory and posterior sensory cortices for the purpose of gating decisions. Dayan [78] also includes a match detection function to allow nonmatch criteria that do not arise naturally from the associative nature of neural networks, an important consideration for our future development of these models.

The models presented here are also generally consistent with the most well-developed models in this domain, procedural models such as ACT-R [60], from which our approach draws inspiration. While our work is generally compatible, we hope to provide more constraints on these theories by considering the wealth of data on detailed aspects of neural function.

In particular, our learning neural network approach will also allow us to constrain theories of exactly what representations are used to produce cognitive sequences by how they are learned. By studying learning over a large number of tasks, we aim to address the question of how these representations emerge on a developmental time scale from a young infant to the fully developed capability of an adult. This focus addresses *learning to learn*, a phenomenon that has both been extensively studied in psychology as well as in machine learning and robotics [79–81]. In both cases, learning to learn transfers beneficial information from a group of tasks to new ones, speeding up learning of new tasks. While in machine learning, many different algorithms have been proposed to achieve transfer learning or learning to learn, a good proportion is based upon representational transfer [79, 82]; that is, due to the efficient and general representations learned in prior tasks, new tasks can be learned more rapidly or more effectively instructed.

To address these questions, we will draw on our and others' work on learning of abstract categories from sensory data (e.g., [83]). Generalizing from prior learning usefully categorizes novel sensory inputs through neural processing that is now relatively well understood. Such category generalization, when combined with the models presented here, offers one explanation of learning to learn. When strategic cognitive sequencing is performed based upon categorical representations (e.g., substitute “input A” in the instructed learning model for “signal to stop and wait for instructions”), learning will generalize to new sensory inputs that can be correctly categorized. This type of generalized matching bears a resemblance to the variable matching rule in recent versions of ACT-R (e.g., “if the word was (word X, previously stored), press the red button”). Modeling this process in greater neural detail will provide more constraints on what types of generalization and matching can be learned and performed by realistic neural networks.

Perhaps because such high-level cognition inevitably involves interactions between many brain regions, computational modeling and other forms of detailed theory construction have, as yet, made little progress. However, the enormous accumulation of work aimed at understanding the contributions from individual brain areas have rendered this complex but important domain a potentially productive target for detailed modeling and computational-level theory.
